# Effects of creep feeding and supplemental glutamine or glutamine plus glutamate (Aminogut) on pre- and post-weaning growth performance and intestinal health of piglets

**DOI:** 10.1186/2049-1891-4-29

**Published:** 2013-08-03

**Authors:** Rafael A Cabrera, James L Usry, Consuelo Arrellano, Eduardo T Nogueira, Marianne Kutschenko, Adam J Moeser, Jack Odle

**Affiliations:** 1 Laboratory of Developmental Nutrition, Department of Animal Science, North Carolina State University, 101 Polk Hall, North Carolina State University, Raleigh, NC 27695, USA; 2 Ajinomoto Heartland Lysine, Chicago, IL 60631, USA; 3 Department of Statistics, North Carolina State University, Raleigh, NC 27695-8203, USA; 4 Ajinomoto do Brasil. Ajinomoto Animal Nutrition, São Paulo, SP 04015-001, Brazil; 5 Department of Population Health and Pathobiology, College of Veterinary Medicine, North Carolina State University, Raleigh, NC 27695, USA; 6 Author current employment: Huvepharma USA, 525 West Park Drive Suite 230, Peachtree City, GA 30269, USA; 7 Author current employment: Micronutrients, 1550 Research Way, Indianapolis, IN 46231, USA

**Keywords:** Creep feeding, Glutamine, Growth performance, Intestinal health, SEM, Villi

## Abstract

**Background:**

Creep feeding is used to stimulate piglet post-weaning feed consumption. L-Glutamine (GLN) is an important source of fuel for intestinal epithelial cells. The objective of this study was to determine the impact of creep feeding and adding GLN or AminoGut (AG; containing glutamine + glutamate) to pre- and post-weaning diets on pig performance and intestinal health. Litters (N = 120) were allotted to four treatments during 14–21 d of lactation: 1) No creep feed (NC, n = 45); 2) creep fed control diet (CFCD, n = 45); 3) creep fed 1% GLN (CFGLN, n = 15); 4) creep fed .88% AG (CFAG, n = 15). After weaning, the NC and CFCD groups were sub-divided into three groups (n = 15 each), receiving either a control nursery diet (NC-CD, CFCD-CD) or a diet supplemented with either GLN (NC-GLN, CFCD-GLN) or with AG (NC-AG, CFCD-AG). Litters that were creep fed with diets containing GLN or AG also were supplemented with those amino acids in the nursery diets (CFGLN-GLN, CFAG-AG). Glutamine was added at 1% in all three post-weaning diet phases and AG was added at .88% in phase 1 and 2 and at .66% in phase 3.

**Results:**

Feed conversion (feed/gain) showed means among treatment means close to significance (*P* = 0.056) and Tukey’s test for pairwise mean comparisons showed that Pigs in the CFGLN-GLN group had the best feed conversion (feed/gain) in the first three-week period post-weaning, exceeding (*P* = 0.044) controls (CFCD-CD) by 34%. The NC-AG group had (*P* = 0.02) the greatest feed intake in the last three week of the study, exceeding controls (CFCD-CD) by 12%. CFGLN-GLN, CFCD-GLN and sow reared (SR) pigs had the greatest (*P* = 0.049) villi height exceeding the CFCD-AG group by 18%, 20% and 19% respectively. The CFAG-AG group had the deepest (*P* = 0.001) crypts among all treatments. CFGLN-GLN, CFCD-GLN and SR groups had the greatest (*P* = 0.001) number of cells proliferating (PCNA) exceeding those in the NC-CD group by 43%, 54% and 63% respectively. Sow reared pigs showed the greatest (*P* = 0.001) intestinal absorption capacity for xylose and mannitol.

**Conclusion:**

Supplementation of creep feed and nursery diets with GLN and/or AminoGut in the first three week improved feed conversion possibly due to improved intestinal health.

## Background

After pigs are weaned from their dams, morphological and functional changes occur in their small intestine. Pluske et al. [1] reported decreased villi height and increased crypt dept. Because newly-weaned pigs are transitioned from milk to dry feed, the pig’s intestinal tract is unable to fully digest and absorb the more complex plant-based macronutrients in the feed. Various researchers [2,3] have reported that this accumulation of undigested and unabsorbed feed creates the perfect medium for opportunistic bacteria such as haemolytic *E. coli* to grow. The normal weaning process stimulates pancreatic development and its enzymatic output; however there is a delay until the different enzymes reach sufficient levels [4]. This in turn can cause post-weaning diarrhea. Creep feeding is deemed to be very important during the suckling period for swine practitioners because it (a) increases weaning weight when offered in small and frequent quantities and (b) eases the transition period for the piglets from sow’s milk to the dry feed. The latter has a physiological implication in order to avoid digestive upset such as post-weaning diarrhea and poor growth. Some argue [5,6] that the use of creep feed during the suckling period could potentially trigger hypersensitivity to feed antigens that can stimulate post-weaning diarrhea. Barnett et al. [7] observed antibody titers in the blood of weaned piglets and confirmed that feed antigens can induce an immune reaction in creep-fed pigs. The reduction in feed intake associated with weaning has been known to affect intestinal integrity and potentially cause pathological disorders. Klasing [8] argued that dietary supplementation of some nutrients or immune modulators can rectify the intestinal impairment and modulate the immune function of animals contributing to improvements in overall health and performance. Nutrition can regulate the type of immune response by a number of mechanisms [8]. Swine nutritionists have traditionally focused on those amino acids that cannot be synthesized by the animals with little attention given to those that can be synthesize by the animals and yet have a great impact on regulating nutrient metabolism and the immune responses [9,10]. These amino acids include arginine, glutamine, glutamate, proline, leucine, cysteine and tryptophan. Recent studies indicate that these amino acids serve important regulatory functions in nutrient metabolism, protein turnover, and immune function, thereby enhancing growth and feed efficiency in pigs. The underlying mechanisms include activation of nitric oxide, mammalian target of rapamycin (mTOR), gaseous signaling, and AMP-activated protein kinase pathways as well as anti-oxidative [11].

Glutamine is a major metabolic fuel for rapidly dividing cells, including enterocytes and lymphocytes, as well as a key regulator of gene expression and cell signaling pathways [12]. Schrock and Goldstein [13] reported that glutamine serves as precursor for the increased renal ammoniagenesis during chronic metabolic acidosis. The amide nitrogen of glutamine is essential for purine and pyrimidine biosynthesis.

Glutamine has important and unique metabolic functions, and it is considered a conditionally essential amino acid in some species under inflammatory conditions [14] and disease states [15,16]. Souba and others [17] have indicated that the provision of GLN-enriched diets in various stress states associated with bacterial translocation decreases the incidence of translocation of bacteria by decreasing the adherence of bacteria to enterocytes. Reeds and others [18] argued that the high metabolic rate of the intestinal mucosa is very unique when compared to the others organs in the body. First, the enterocytes are specialized in absorbing nutrients from the lumen to the basal lamina. Second, mucosa cells are presented with high quantities of substrates from both the intestinal lumen and the mesenteric arterial circulation. Accordingly, under fed conditions, the quantification of substrate used by the gut can be challenging to quantify given the dual supply from both diet and the arterial circulation. Finally, GLN is the only amino acid in arterial blood that is taken up by the small intestine in swine [19]. The small intestine (even though only represents 3 to 4% of the body weight) utilizes 30% of the arterial GLN and 67% of dietary GLN in swine. For comparison, 95 to 97% of dietary glutamate is extracted by the pig small intestine in first pass [20,21] but only 50% is metabolized to CO_2_[18].

Because the gastrointestinal tract has an obligatory requirement for L-GLN [18] and availability of L-GLN from endogenous tissue production may not be sufficient for the maintenance of the structural and functional integrity of the intestinal mucosa [22,23], We hypothesized that creep feeding of suckling piglets and adding L-glutamine or the combination of L-glutamine and L-glutamate to pre and/or post-weaning diets would alleviate villi atrophy, reduce post-weaning diarrhea and improve post-weaning growth.

The objective of the study was to evaluate the effects of L-GLN and AminoGut (containing L-GLN and L-glutamate) on intestinal histology, intestinal absorptive capacity, enzymatic activity, and growth performance in a commercial swine operation. The effects of these supplements on piglet growth performance have not been evaluated during the whole nursery period following supplementation during the pre-weaning period.

## Methods

**Figure 1:**
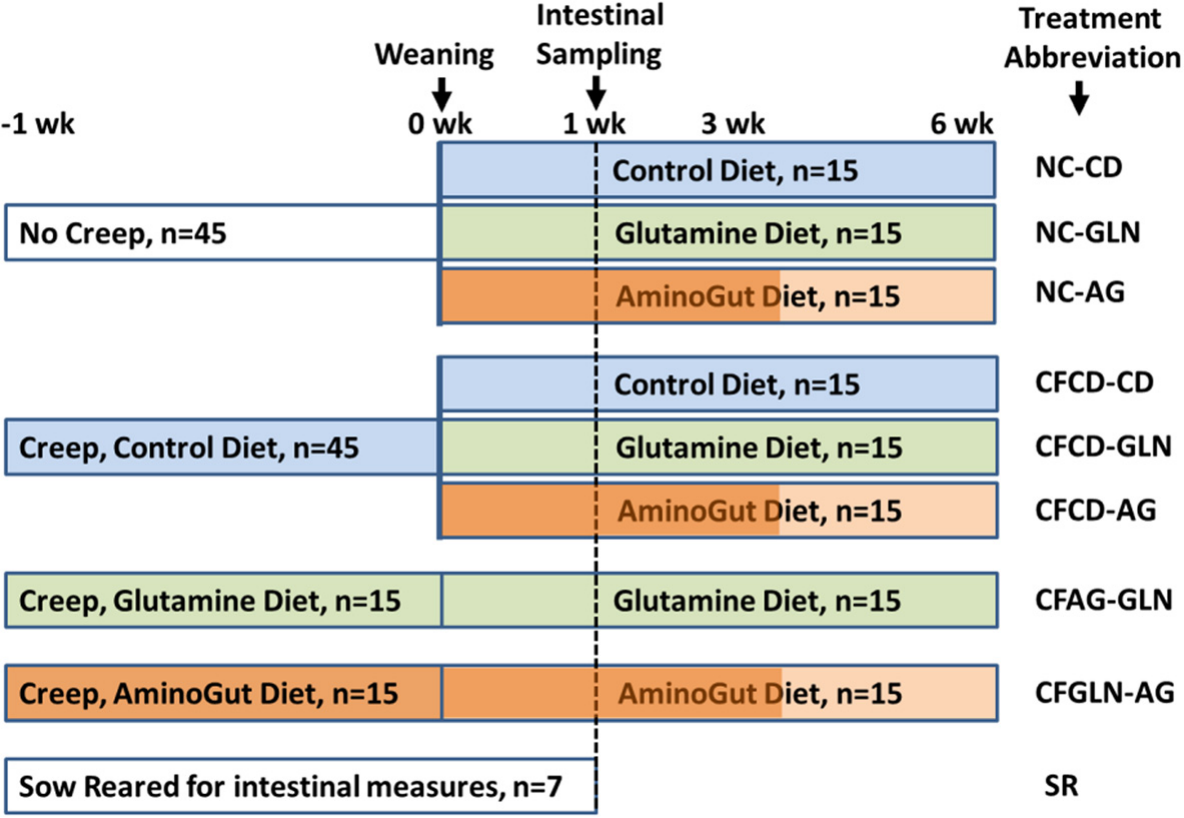
**Experimental design showing four pre-weaning creep diet groups and eight post-weaning diet groups together with sow-reared control pigs.** Creep feed was initiated 1 week prior to weaning and consisted of pelleted Phase 1 diets. Post-weaning diets consisted of either control basal diet (see Table 1) with additions of either 1% L-GLN (in all feed Phases 1–3) or 0.88% AminoGut in Phases 1 and 2 and 0.66% in Phase 3. Pigs were weighted at birth, weaning, (some at 1 wk post-weaning), 3 wk and 6 wk post-weaning. Selected pigs were euthanized (n = 7/trt) at 1 wk post-weaning for intestinal health measurements. Various treatment abbreviations are also summarized.

All protocols were under the supervision of licensed veterinarians. Standard operating procedures for animal care were in accord with published guidelines for animal care [24]. The experimental animals were not subjected to prolonged constraint or surgical procedures and were humanely treated throughout the experiment. The study was conducted during the summer 2010 on a 4800-sow farm in Maple Hill, NC (Murphy-Brown, LLC; Rose Hill, NC). One-hundred and twenty litters were randomly allotted to one of eight dietary treatment scenarios (Figure 1). At one week prior to weaning, four creep-feed treatments were initiated: A) No creep feed; B) Creep feed, control diet; C) Creep feed containing 1% GLN; D) Creep feed containing 0.88% AminoGut. AminoGut is a commercial dietary supplement produced by Ajinomoto do Brazil (São Paulo, Brazil) containing a mixture of L-glutamine (min 10%) and L-glutamate (min 10%). The pelleted creep feed was a phase 1 nursery diet (Table 1), manufactured at the North Carolina State University feed mill. Litters were offered fresh creep feed at 4-h intervals from 8 am to 4 pm each day. Litters weights were recorded at birth (WayPig model 252, Raytec Manufacturing, Ephrata, PA) and weaning (Avery Weight-Tronix model 640, http://www.agscales.com, Fairmont, MN). Pigs were weaned at an average of 21 d and were transported to the Site 2 nursery (~ 300 meters from farrowing Site 1), and litters from pre-weaning treatments A and B were each divided among the following nursery diets: 1) Control diet, 2) GLN diet, and 3) AminoGut diet (Figure 1, Table 1). Litters from pre-weaning treatments C and D were continued on similar diets post-weaning (e.g., GLN and AminoGut respectively). Additional litters were allowed to nurse the sow (without creep feed) until 4 week of age to provide age-matched controls for invasive measures of intestinal health (n = 7). The experimental design is illustrated in Figure 1, showing abbreviations used for each dietary treatment scenario. After weaning, phase 1 diet (Table 1) was budgeted at 2.72 kg /pig, Phase 2 diet was budgeted at 5.45 kg/pig and Phase 3 diets was budgeted at 18.16 kg /pig. At 3 and 6 week postweaning, the pigs and feeders were weighed for growth and feed conversion calculations.

**Table 1:** **Ingredients and nutrient composition of the basal diets (Phase 1, 2 and 3)**^**1**^

**Ingredients, %**	**Phase 1**	**Phase 2**	**Phase 3**
Corn	39.00	51.36	63.73
Soybean meal, 48%	20.00	25.00	30.00
Choice white grease	3.00	3.00	3.00
Dicalcium	0.00	0.60	1.30
Limestone	0.55	0.60	0.50
Salt	0.30	0.30	0.50
l-Lysine	0.25	0.27	0.39
DL-Methionine	0.21	0.18	0.18
L-Threonine	0.16	0.16	0.19
L-Tryptophan	0.02	0.02	0.00
Granular whey	5.00	0.00	0.00
Porcine plasma protein	4.00	2.00	0.00
Dairy Lac 80	20.00	10.00	0.00
Fish meal select	6.00	5.00	0.00
ZnO	0.30	0.30	0.00
Copper sulfate	0.05	0.05	0.05
Vitamins Mix^2^	0.01	0.01	0.01
Trace mineral Mix^3^	0.15	0.15	0.15
Mecadox	1.00	1.00	0.00
**Total**	**100.00**	**100.00**	**100.00**
**Calculated nutrients**			
ME (Mcal/kg)	3.52	3.46	3.46
CP, %	21.67	21.77	20.12
TID Lysine, % ^4^	1.39	1.35	1.25
TID Met, %	0.52	0.50	0.45
TID M + C, %	0.83	0.81	0.75
TID Threonine, %	0.93	0.88	0.82
TID Tryptophan, %	0.25	0.25	0.21
TID Isoleucine, %	0.77	0.78	0.73
TID Valine, %	0.93	0.91	0.82
Glutamate, %	3.69	3.79	3.62
Total P, %	0.64	0.67	0.63
Available P, %	0.43	0.40	0.30
Ca, %	0.80	0.80	0.60
Na, %	0.32	0.21	0.21
Lactose, %	19.50	8.00	0.00

^1^L-Glutamine was supplemented at 1% of the diet at the expense of corn. Amino-Gut was supplemented 0.8% in phase 1 and 2; and at 0.6% in phase 3.

^2^Vitamin Mix contained per kg of premix: vitamin A Acetate 4,231,292 IU; vitamin D_3_ 603,174 IU; vitamin E ascorbate 24,172 IU; vitamin B_12_ 14.97 mg; riboflavin spray dried 3,023 mg; niacin 18,140 mg; d-pantothenic 12,093 mg; menadione 1,995 mg; folic acid spray dried 907 mg; biotin spray dried 121 mg.

^3^Trace mineral Mix contained: ferrous sulfate 11.02%; copper sulfate 1.10%; ethylenediamine dihydriodine 198 ppm; manganese oxide 2.64%; sodium selenite 198.4 ppm; zinc sulfate 11.02%.

^4^Total ileal digestible lysine.

At one week post weaning, one pig per pen was fasted overnight and then intra-gastrically gavaged with a D-xylose/mannitol solution as follows. A solution containing 0.2 g/L of D-xylose (Pfizer, N.Y., NY) and 0.3 g/L of mannitol (Sigma, Saint Louis, MO) was prepared and was given to the pigs on an average of 9.5 h after fasting. The selected dose was 6.5 mL/kg of body weight. Pigs were individually weighed (Berkley FS-50 hanging scale, Somers Point, NJ). At precisely two h post gavage, pigs were bled via jugular venipuncture. The time of bleeding was selected based upon work by Doerfler et al. [25]. After pigs were bled they were humanly euthanized for collection of intestinal tissues. Jejunum samples (25 cm from the stomach) were collected for both light and scanning electron microscopy (SEM). The portion cut specifically for SEM, was cut open and laid flat in a small cartridge in order to obtain a better picture of the intestinal villi. A separate jejunum tissue sample was cut and the intestinal mucosa was scrapped for maltase activity analysis. Blood was centrifuged after 24 h and the serum stored at −20°C for further analysis. Performance data were statistically analyzed using the PROC GLIMMIX of SAS with birth weight and weaning age as covariates. Intestinal and serum metabolite data were analyzed using the Mixed Procedure of SAS with the body weight (one week post-weaning in the nursery) used as covariate.

### Scanning electron microscopy

Jejunum samples were collected from four-week old piglets (1 wk post-weaning) and immersed in 4 F:1G fixative containing 4% formaldehyde and 1% glutaraldehyde in a phosphate buffer, with an osmolarity of 176 mOsM and a pH of 7.2-7.4 [26]. Samples were cut to be between 2–3 mm in order to minimize chances of bulk charging. Samples were rinsed in 0.1 mol/L phosphate buffer and dehydrated in an ethanolic series to 100% ethanol before subjection to critical point drying after being stored for approximately 7 wk in the 4 F: 1G fixative. Samples were then mounted on SEM stubs with carbon tape and sputter coated with gold-palladium before being viewed with a JEOL JSM-6360LV scanning electron microscope (JEOL, Peabody, MA). This microscope is a fully digital instrument that can view specimens by secondary electron imaging (SEI), backscatter electron imaging (BEI), at high vacuum, or at low vacuum.

### Hematoxylin & eosin staining

Jejunum samples were collected (25 cm from the stomach) and preserved in a formalin solution and stored in room temperature for histology analysis. Tissues were trimmed into five millimeter thick sections and placed in processing cassettes. The tissues were processed in a Tissue-Tek VIP5 tissue processor (Sakura Finetek, Torrance, CA) using a standard overnight processing schedule. Tissues were embedded in paraffin and five micron sections were mounted on glass slides. The slides were stained on a DRS-601 slide stainer (Sakura Finetek, Torrance, CA) with hematoxylin and eosin, cleared and mounted with a permanent media. The stained tissues on glass slides were examined using an Olympus AH-2 Vanox-S microscope (Ultrasonic Power Corporation, Freeport, IL) and measured using SPOT™ software (SPOT™ Imaging Solutions, Sterling Heights, MI).

### PCNA Staining

Five micron jejunal slices were mounted on glass slides. A primary mouse monoclonal antibody (PC10) was used for the as a proliferation marker. This antibody is specific for proliferating cell nuclear antigen, PCNA, p36 protein expressed at high levels in proliferating cells. It was diluted at 1:1,500 and incubated for 30 min. The remaining steps were completed using the Dako EnVision Mouse kit (Dako, Denmark). Intensively stained and the total number of enterocytes were counted in 8 consecutive well-orientated crypts (those that extended to the muscularis mucosa).

### Analysis of mannitol

Samples of serum were frozen, thawed at room temperature and vortexed to mix. Samples were then filtered by centrifugation using Ultrafiltration Spin Columns (0.45 μm, Millipore, Temecula, CA). An aliquot of 200 μL of sample was transferred to HPLC autosampler vials containing 250 μL inserts. An internal standard solution of myo-inositol was added (2 μL). Analysis was done using High Performance Liquid Chromatography (HPLC). The extracts were analyzed using a Dionex BioLC (Dionex Corporation, Sunnyvale, CA) at a controlled temperature of 30°C. The system consisted of a gradient pump, an autosampler, and a pulsed amperometric detector. The mobile phase was 52 mmol/L NaOH (Thermo-Fisher Chemical Corp. Pittsburgh, PA) at an isocratic flow rate of 1.0 mL/min. The column used was a Dionex PA-10, 250 mm length and 4 mm i.d., fitted with Dionex PA-10 and borate guard columns. The detector was programmed to run a quadruple waveform as recommended by the manufacturer. A shift in the detector range was 1 μC. The injection volume was 10 μL. The mannitol was calculated using an authentic standard of d-mannitol and myo-inositol as an internal standard. All the reference standards were purchased from Sigma Chemical Corp (St. Louis, MO).

### Analysis of xylose

The collected pig serum (20 μL) was subjected to a modified micro method [27,28] first described by Eberts et al. [29] for determination of plasma D-xylose. To each 20 μL plasma sample, 2 mL of phloroglucinol (Sigma Chemical Co., Saint Louis, MO 63178–9916) color reagent was added and heated for 4 min at 100°C. The samples were allowed to cool at room temperature in a water bath. After cooling, the absorbance of each sample was read on a Gilford UV–vis spectrophotometer (Thermo Fisher Scientific, Inc.; Waltham, MA) set at 554 nm.

### Maltase enzyme activity

The maltase assay was performed as described by Dahlqvist [30]. Maltase activity (U/g of protein) was expressed as units, with 1 unit defined as the amount of enzyme transforming 1.0 μmol of substrate per min at 25°C.

## Results

**Figure 2:**
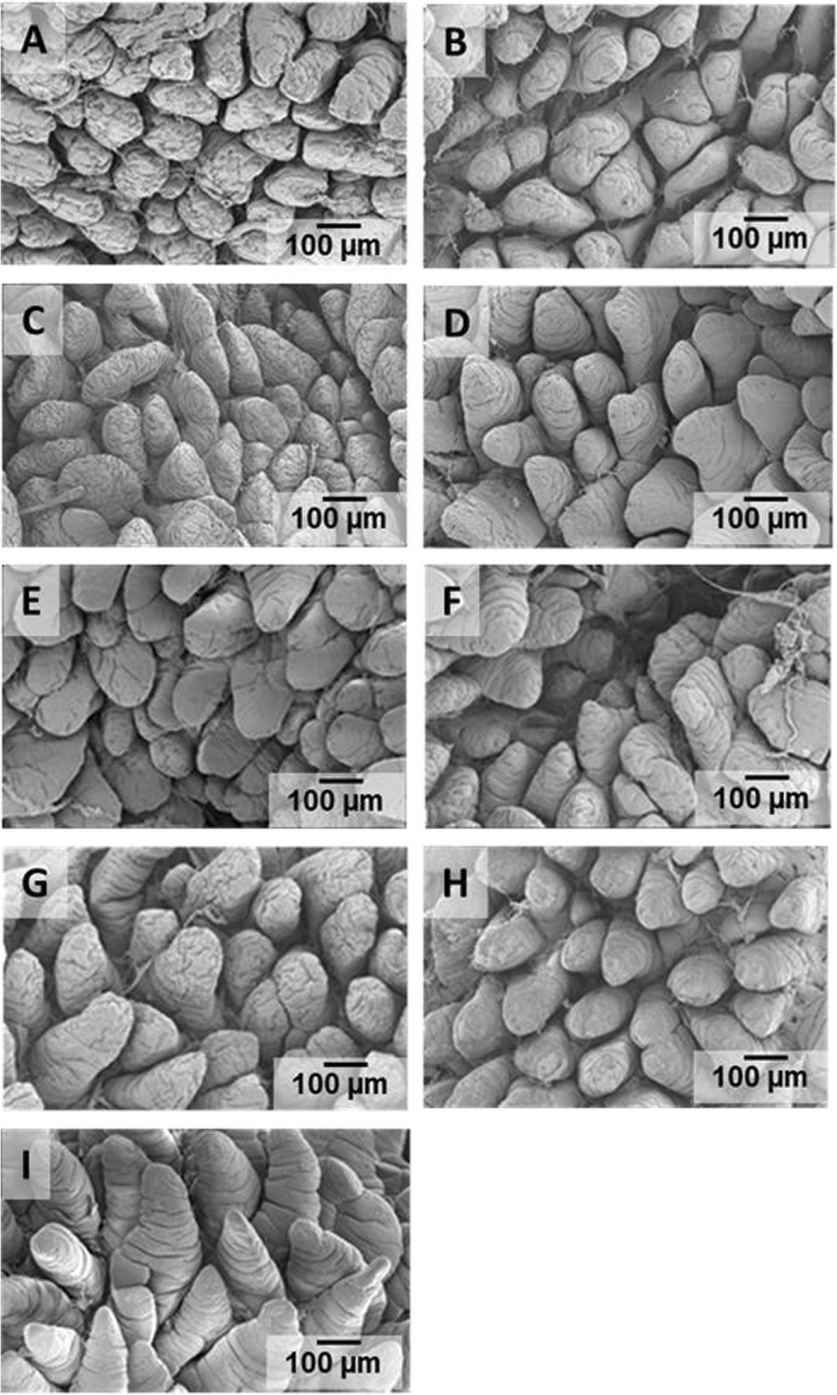
**Scanning electron micrographs of jejunal villi of pigs at four wk of age. A**. No creep Control Diet (NC-CD). **B**. No creep Glutamine (NC-GLN). **C**. No creep AminoGut (NC-AG). **D**. Creep Fed Control Diet-Control Diet (CFCD-CD). **E**. Creep Fed Control Diet-Glutamine (CFCD-GLN). **F**. Creep Fed Control-AminoGut (CFCD-AG). **G**. Creep Fed Glutamine-Glutamine (CFGLN-GLN). **H**. Creep Fed AminoGut-AminoGut (CFAG-AG). **I**. Sow-Reared Control.

Results for pre-weaning performance are summarized in Table 2. We found weaning age to be significant (*P =* 0.001) among the pre-wean treatments. For subsequent comparisons, this variable was used as covariate. Pigs/litter, sow parity, birth weight, weaning weight, and mortality were not different among the treatments. Creep feed consumption also did not differ for those treatments receiving creep feed. The average creep feed consumptions for the control diet and those supplemented with either glutamine or AminoGut were 49.44, 45.57 and 48.44 g/pig respectively. We did not find an effect of creep feeding on post-weaning performance (Tables 3 &4). A longer (> 1 wk) creep feed period needs to be examined. Feed conversion (feed/gain) showed means among treatments close to significance (*P* = 0.056) and Tukey’s test for pairwise mean comparisons showed that Pigs in the CFGLN-GLN group had the best feed conversion (feed/gain) in the first three-week period post-weaning, exceeding (*P* = 0.044) controls (CFCD-CD) by 34%. All others variables were not significant during this post-weaning period among the treatments (Table 3). The NC-AG group had (*P* = 0.02) the greatest feed intake among all treatments in the last three week of the study (Table 4), exceeding controls (CFCD-CD) by 12%. All others variables were not significant during this post-weaning period among the treatments. CFCD-GLN, Sow-Reared and CFGLN-GLN groups had the greatest (*P* = 0.049) villi height exceeding those which were creep fed with a control diet and later supplemented with AminoGut (CFCD-AG) by 20%, 19% and 18% respectively (Table 5). The Sow-Reared group was added as a point of reference against the other treatments. All tissue samples for all treatments were taken at 28 d of age. We also found that pigs creep fed with a diet supplemented with AminoGut and fed a post-weaning diet supplemented with AminoGut (CFAG-AG) had the deepest (*P* = 0.001) crypts among all the treatments. Sow-Reared, CFCD-GLN and CFGLN-GLN, and groups had the greatest (*P* = 0.001) number of cells proliferating (PCNA), exceeding those which did not receive creep feed and later receiving a control diet (NC-CD) by 63%, 54% and 43% respectively. We found a correlation between villi height and PNCA: the taller the villi height, the greater the number of proliferating cells. Sow-Reared pigs showed the greatest (*P* = 0.001) intestinal absorption capacity for xylose and mannitol when compared with the others treatments. The levels of xylose and mannitol found in the sow reared pigs blood exceeded the average of the levels found in the other treatments by 3.2 and 7.4 folds respectively. This is consistent with the architecture of the villi of the sow reared pigs when compared to the other treatments (see qualitative SEM images, Figure 2). There was no significance difference among the other treatments on the absorption of these sugars. We found the levels of xylose in the blood to be higher than those of mannitol even though a higher amount of mannitol was diluted in the final solution (0.2 g/L vs. 0.3 g/L). We found no significant differences among the treatments in maltase activity although there was a tendency (*P* = 0.18) for creep fed treatments to be numerically different than those which did not receive creep feed (260 vs. 214 μmoles / min. g of protein respectively).

**Table 2:** Pre-weaning performance of creep-fed piglets

**Variables**	**No-Creep**	**Creep**	**Creep + Glutamine**	**Creep + AminoGut**	**SEM**	***P *value**
No. litters	45	45	15	15	-	-
Wean age, d	21.5	20.5	20.3	18.9	0.13	0.001
Pigs/litter	12.5	12.4	12.5	11.9	0.23	0.286
Sow parity	3.4	3.4	3.4	3.4	0.20	0.998
Birth wt, kg/pig	1.5	1.4	1.4	1.5	0.04	0.250
Wean wt, kg	6.5	6.4	6.4	6.5	0.26	0.980
Creep feed, g/pig^1^	-	49.4	45.6	48.4	2.93	0.617
Mortality, pigs/litter	1.0	1.2	1.5	1.2	0.22	0.488

^1^Creep feed was offered for one week prior to weaning.

**Table 3:** Pig performance from week 1 to 3 post-weaning

**Variables**	**NC-CD**^**1**^	**NC-GLN**	**NC-AG**	**CFCD-CD**	**CFCD-GLN**	**CFCD-AG**	**CFGLN-GLN**	**CFAG-AG**	**SEM**	***P *value**
No. pens	6	6	6	6	6	6	6	6	-	-
No. pigs	144	144	144	144	144	144	144	144	-	-
Initial wt, kg	6.50	6.35	6.59	6.14	6.39	6.61	6.42	6.52	0.34	0.984
Final wt, kg	10.4	10.20	10.66	9.51	10.06	10.08	10.65	10.34	0.26	0.128
ADG, kg/pig	0.25	0.24	0.25	0.19	0.23	0.22	0.26	0.24	0.02	0.124
Intake, kg/pig	0.33	0.28	0.30	0.27	0.27	0.28	0.29	0.29	0.02	0.644
Feed/gain	1.35^bc^	1.17^ab^	1.17^ab^	1.48^c^	1.15^ab^	1.30^b^	1.11^a^	1.21^ab^	0.09	0.056
Mortality	0.38	1.43	0.94	1.28	0.80	0.63	0.82	0.73	0.37	0.562
Removed	1.23	0.90	2.00	2.49	1.28	1.03	1.81	0.59	0.70	0.584

^1^*NC-CD* No Creep Control Diet, *NC-GLN* No Creep Glutamine, *NC-AG* No Creep AminoGut, *CFCD-CD* Creep Fed Control Diet-Control Diet.

*CFCD-GLN* Creep Fed Control Diet-Glutamine, *CFCD-AG* Creep Fed Control Diet-AminoGut, *CFGLN-GLN* Creep Fed Glutamine-Glutamine.

*CFAG-AG* Creep Fed AminoGut-AminoGut. See Figure 1.

^abc^Least square means within a row lacking a common superscript are different (*P* < 0.05).

**Table 4:** Pig performance from week 3 to 6 post-weaning

**Variables**	**NC-CD**^**1**^	**NC-GLN**	**NC-AG**	**CFCD-CD**	**CFCD-GLN**	**CFCD-AG**	**CFGLN-GLN**	**CFAG-AG**	**SEM**	***P *value**
No. pens	6	6	6	6	6	6	6	6	-	-
Initial wt, kg	10.45	10.32	10.72	9.44	10.18	10.10	10.78	10.43	0.43	0.457
Final wt, kg	22.32	21.97	22.74	22.21	22.86	22.50	21.71	22.76	0.41	0.437
ADG, kg/pig	0.52	0.51	0.55	0.50	0.54	0.53	0.50	0.54	0.02	0.444
Intake, kg/pig^2^	0.77^b^	0.74^b^	0.86^a^	0.77^b^	0.79^b^	0.79^b^	0.70^c^	0.80^b^	0.03	0.022
Feed/gain^2^	1.39	1.48	1.65	1.49	1.64	1.84	1.66	1.50	0.12	0.271
Mortality,%	0.01	0.00	0.35	0.13	0.16	0.01	0.19	0.01	0.13	0.434
Removed,%	1.01	1.5	1.69	1.62	0.99	1.15	1.69	1.17	0.32	0.497

^1^*NC-CD* No Creep Control Diet, *NC-GLN* No Creep Glutamine, *NC-AG* No Creep AminoGut, *CFCD-CD* Creep Fed Control Diet-Control Diet, *CFCD-GLN* Creep Fed Control Diet-Glutamine, *CFCD-AG* Creep Fed Control Diet-AminoGut, *CFGLN-GLN* Creep Fed Glutamine-Glutamine, *CFAG-AG* Creep Fed AminoGut-AminoGut. See Figure 1.

^2^For the calculation of feed intake and feed/gain, the number of pens was reduced from 6 to 3 pens because two pens shared a common feeder.

^abc^Least square means within a row lacking a common superscript are different (*P* < 0.05).

**Table 5:** Intestinal morphology, PCNA staining, and maltase activity, and serum xylose and mannitol following oral gavage of pigs 1-wk post-weaning

**Variables**	**NC-CD**^**1**^	**NC-GLN**	**NC-AG**	**CFCD-CD**	**CFCD-GLN**	**CFCD-AG**	**CFGLN-GLN**	**CFAG-AG**	**Sow Reared**	**SEM**	***P* value**
No. pigs	6	6	6	6	6	6	6	6	6	-	-
Age pigs, d	28	28	28	28	28	28	28	28	28	-	-
Body wt, kg^2^	6.5	6.7	6.2	5.8	6.4	6.4	6.5	6.5	6.3	0.28	0.824
Villi height, μm	508^ab^	515^ab^	510^ab^	560^ab^	576^a^	480^b^	568^a^	560^ab^	570^a^	23	0.049
Villi width, μm	136	140	120	133	148	158	145	147	132	8.9	0.301
Crypt depth, μm	202^ab^	188^b^	177^b^	192^ab^	218^ab^	242^ab^	225^ab^	258^a^	118^c^	14	0.001
PCNA, cells	56^c^	81^a^	67^b^	71^ab^	86^a^	70^ab^	80^a^	64^b^	91^a^	5.5	0.001
Xylose, mg/dl	14.6^b^	13.5^b^	11.1^b^	14.4^b^	9.5^b^	14.1^b^	15.4^b^	13.7^b^	42.8^a^	1.94	0.001
Mannitol, mg/dl	1.8^b^	1.5^b^	2.3^b^	1.9^b^	2.8^b^	3.0^b^	2.8^b^	3.0^b^	17.7^a^	0.68	0.001
Maltase, μmol/(min. g protein)	256	183	201	299	282	254	244	242	239	32	0.189

^1^*NC-CD* No Creep Control Diet, *NC-GLN* No Creep Glutamine, *NC-AG* No Creep AminoGut, *CFCD-CD* Creep Fed Control Diet-Control Diet.

*CFCD-GLN* Creep Fed Control Diet-Glutamine, *CFCD-AG* Creep Fed Control Diet-AminoGut, *CFGLN-GLN* Creep Fed Glutamine-Glutamine.

*CFAG-AG* Creep Fed AminoGut-AminoGut. Sow Reared were pigs kept with the sow until they were 28 d of age. See Figure 1.

^2^ Body weight was taken one week post-placement in the nursery. Sow reared pigs stayed with their dams for 28 d.

^abc^ Least square means within a row lacking a common superscript are different (*P* < 0.05).

## Discussion

Windmueller and Spaeth [31] determined that in the adult rat small intestine, CO_2_, lactate, alanine and glucose account for 56–64, 16–20, 4–8, and 2-10% of the total catabolized carbons of luminal glutamine, glutamate and aspartate, respectively. These results and others showed that amino acids (glutamine, glutamate and aspartate), rather than glucose, are the major fuels for the small intestine mucosa, responsible for providing energy required for intestinal ATP-dependent metabolic processes [32]. Although there seems little doubt that glutamine plays an important, but remarkably poorly characterized role in the metabolism of many proliferating cells, much of the more recent literature on intestinal metabolism has ignored two observations made by Windmueller and Spaeth [33]. Those are, first, that the metabolism of luminal glutamate was even more extensive than that of arterial glutamine; and second, that the presence of high concentrations of glutamate in the intestinal lumen had only a small (less than 25%) effect on intestinal utilization of glutamine. This perhaps suggests that these two closely related amino acids may have different functional roles in the intestinal mucosa.

It seems that glutamate can partially substitute for Gln in several pathways, including ATP production and synthesis of arginine, alanine, proline and aspartate [34]. Glutamate plays a significant role on avoiding Gln degradation by mitochondrial phosphate-activated glutaminase in extra hepatic tissues and cells yielding a sparing effect on the use of Gln as a metabolic fuel [35] and its availability in cells [36]. Wu [10] rightly notes that that key functions of Gln (syntheses of Gln-tRNA, aminosugars, carbamoylphosphate, NAD, NADP, as well as purines and pyrimidines; renal ammoniagenesis; and regulation of ornithine decarboxylase expression) cannot be supplied by glutamate. Wu and others [37] argued that although both Gln and glutamate provided in the enteral diet are extensively catabolized by the small intestine, this organ takes up Gln, but not glutamate, from the arterial blood. They suggested that due to the complex compartmentalization of cellular metabolism, extracellular glutamate may channel preferentially into the cytoplasm rather than into the mitochondria and, therefore, have different effects than the glutamate generated from Gln in mitochondria.

The vast majority of research showing the benefits of supplementing Gln in the diet can be found in research with swine. Wu and co-workers [38] reported that among all the amino acids, uterine and umbilical uptake of Gln was the greatest in pregnant gilts, implicating an important role for Gln in fetal growth and development. They fed 1% Gln in the diet of gestating gilts between 90 and 114 d of gestation and found that it significantly increased average birth weight. They also found that the number of intrauterine growth retarded piglets, variation in birth weight and pre-weaning mortality were reduced by 39, 33, and 46%, respectively, when compared with the control group.

Kim and Wu [39] reported that lactating sows have a high requirement for Gln and the uptake of Gln by the porcine mammary glands may be inadequate for the synthesis of milk proteins. By d 10 during the lactating period, the mammary glands uptake 16 g Gln/d from the arterial circulation [40], however Haynes and others [41] reported that at that point in time, 36 g Gln/d is being secreted. Wu and colleagues [38] fed 1% Gln from d1 to d 20 to lactating sows and found an increase of Gln concentrations in the plasma, skeletal muscle and whole milk of the sows, as well as piglet growth and survival.

Haynes et al., [41] evaluated the effectiveness of Gln or L-alanyl-L-glutamine (Ala-Gln) *in vivo* with 7-d-old piglets challenged with a single intraperitoneal injection LPS (0.1 mg/kg body weight). Administration of Gln or Ala-Gln to LPS challenged piglets increased Gln concentrations in small intestinal lumen and plasma, reduced intestinal expression of TLR-4, active caspase-3 and NF*k*B, ameliorated intestinal injury, decreased rectal temperature and enhanced growth performance. These results demonstrate a protective effect of Gln or Ala-Gln against LPS-induced enterocytes death. They also reported that the Gln supplementation stimulated the growth of sow-reared piglets by 12%.

Yi et al. [42] found that feeding glutamine had beneficial effects in alleviating growth depression of *E. coli* K88^+^-challenged pigs, mainly via maintaining intestinal morphology and function, and/or possible modulating the somatotrophic axis. Jiang and others [43] reported similar results. Wu et al. [44] orally administered Gln (0.5 g/kg BW/d) to low-birth weight piglets from 0 to 21 d of age and found that their growth were improved by 16% and their pre-wean mortality by 48%.

Our results are most consistent with those reported by Wu et al., [45]. They found a 29% improvement in feed conversion (21d post-weaning) when supplementing 1% glutamine. Glutamine (Gln) supplementation (1%) prevented jejunal atrophy (measured as villus height) during the first week post-weaning and increased feed: gain ratio (indicator of growth performance) by 25% during the second week post-weaning. It also increased plasma concentration of aspartate, glutamate and alanine and also reduced the extent to which plasma taurine concentration fell in post-weaning pigs. The prevention of villi atrophy during the first week post-weaning also has been reported by Wang and co-workers [46].

Liu and others [47] reported similar results than those reported by Wu et al. [45]. They fed 1% L-glutamine or 1% L-glutamate to weaned pigs from 28-d to 42-d of age. Jejunal atrophy was prevented during the first week for the groups fed either L-GLN or L-glutamate when compared to the control group. Again these results provide an experimental basis for the use of glutamine and glutamate to improve piglet intestinal health and to support improved growth performance.

D-Xylose absorption test has been used as a tool for the assessment of the effect of anticoccidials on the intestinal absorptive capacity of broilers during experimental coccidiosis [48] and malabsorption in poult enteritis and mortality syndrome [25]. D-xylose, a poorly metabolized pentose sugar, is well absorbed from the small intestine of chickens and readily excreted in the urine [25]. Blood D-xylose concentrations are expected to peak at 30–60 min after intake in poultry [25,48] and 60 min in pigs [49,50].

Mannitol has been clinically applied in diagnostic and therapeutic doses for 1) the determination of extracellular fluid volume and glomerular filtration rate, 2) testing intestinal absorption and mucosal integrity, 3) as a diuretic and 4) as a radical scavenger and osmotically active component of infusions.

There are few studies using these two sugars as markers of gastrointestinal *in vivo* permeability in pigs in a commercial setting. In this study, the uptake of xylose was greater than mannitol regardless of their molecular weight (150 and 182 g/mol respectively) and the amount administered (mannitol higher than xylose). Xylose can be metabolized in the gut by bacteria, and then absorbed whereas mannitol cannot. Therefore we would expect xylose to be absorbed more rapidly than mannitol. Mannitol is partially metabolized, the remainder being excreted in the urine. Nasrallah and Iber [51] administered orally doses of 20 to 100 g of ^14^C-mannitol to five humans with cirrhosis of the liver and to five subjects who had normal liver function. They found that at least one-sixth of orally ingested mannitol is absorbed and about one-third is metabolized.

The lack of significant differences in performance among the treatments for the entire 6-wk period correlates well with the lack of significant differences among the treatments for the levels xylose and mannitol absorbed and found in their blood. We were not surprised by the high levels of intestinal absorptive capacity shown by the sow reared pigs when compared to the other treatments.

These tests of small intestine permeability to low-molecular-weight carbohydrates have a considerable application in the study of small intestine diseases such as celiac disease in humans [52], diagnosing food allergy and assessing the effectiveness of anti-allergic agents such as sodium cromoglycate [53].

In young animals, lactase activity prevails, however as it gets older then maltase activity (as well amylases, lipases proteases) increases. Low concentration of maltase in the surface of epithelial cells may be an indication of villus atrophy due to disease or malnutrition [54]. We were unable to find any significant differences among the treatments in maltase activity.

Scanning electron microscopy (SEM) allows observation of the surface of the epithelium in three dimensions and gives a fresh dimension in the investigation of gut mucosa [55]. The visual assessment of the SEM graphs showed that pigs which were not creep fed during the suckling period had a rough villi surface with numerous cells shedding (apoptosis) along the entire length of the villi (Figure 2A). They also showed deep transversal furrows in most (if not all) the epithelial cells (Figure 2A, B). Those treatments creep fed either with a control diet or supplemented with glutamine or Aminogut showed longer villus than those treatments which were not creep fed (552 microns vs. 511 microns respectively) (Figure 2 D, E & F). The CFGLN-GLN treatment showed elongated, well defined and high villus (Figure 2G). Increased villus height could increase total luminal villus absorption area and could result in adequate digestive enzyme concentration and/or increased transport of nutrient at the villus surface. Gln has been shown to enhance epithelial repair in several models of intestinal injury and to stimulate epithelial proliferation and protein synthesis or reduce apoptosis in cell culture [56,57]. Increased uptake of Gln in the crypts not only could promote a compensatory increase in Na^+^ absorption but also would place this nutrient in the ideal location to promote crypt cell production and restoration of the villus architecture. The CGAG-AG treatment showed deep and wide crypts. This could be explain by the fact that glutamine is donating an amide group for the biosynthesis of purines (adenine and guanine) and pyrimidines (thymine and cytosine) which are the nucleotides bases to support nucleic acid production (DNA) for rapidly dividing cells in the crypts. In RNA, the complement of adenine is uracil instead of thymine. The sow reared pigs showed what may be the perfect villi structure: healthy, well defined villus, no signs of apoptotic cells and sufficient mucin production (Figure 2I). Mucins are a family of high molecular weight, heavily glycosylated proteins produced by epithelial tissues (specifically by the goblet cells) in most metazoans. Two noticeable jejunal villi structure characteristics in all treatments for 28 d pigs were 1) transversal furrows that were present along the entire length of the villi and 2) the shape of the villi were not finger-like but rather wide and tongue-like in shape. It was evident that the small intestinal mucosa undergoes profound structural and developmental changes during the first 4 week of the pig’s life and these changes are manifested in shape, size and density of the villi.

## Conclusion

The supplementation of glutamine and glutamine plus glutamate (AminoGut) in pre- and post-weaning diets improved feed conversion in the first three week post-weaning when compared to CFCD-CD treatment. These findings are in the agreement with those reporting a reduction in villi atrophy when supplementing glutamine at 1% in diets during the first week post-weaning. Sow reared pigs showed the best intestinal absorptive capacity and villi architecture. More research is needed at the field level to justify the economical feasibility of adding either glutamine or AminoGut in current commercial livestock diets and the European model of weaning pigs at 28 d of age. Consideration should also be given to potential supplementation of the sow to enrich milk concentrations [58].

The existing vast knowledge of the roles of functional AA’s such as glutamine and others (arginine, glutamate, proline, leucine, cysteine and tryptophan) provides the scientific basis for nutritionists to revise current nutrient requirements for livestock especially weaned pigs. These findings indicate that strong consideration must be given to GLN and glutamate as nutritionally essential amino acids for post-weaning pigs diets.

## Abbreviations

mTOR: Mammalian target of Rapamycin; IgA: Immunoglobulin A; GLN or Gln: Glutamine; AG: AminoGut; NC: Non-Creep fed; CFCD: Creep Fed Control Diet; CFGLN: Creep Fed Glutamine; CFAG: Creep Fed AminoGut; NC-CD: Non-Creep Fed and later receiving a Control Diet; CFCD-CD: Creep Fed Control Diet and later receiving a Control Diet; NC-GLN: Non-Creep Fed and later receiving a diet supplemented with Glutamine.; CFDC-GLN: Creep Fed Control Diet and later receiving a diet supplemented with Glutamine.; NC-AG: Non-Creep Fed and later receiving a diet supplemented with AminoGut; CFCD-AG: Creep Fed Control Diet and later receiving a diet supplemented with AminoGut.; CFGLN-GLN: Creep Fed Glutamine and later receiving a diet supplemented with Glutamine.; CFAG-AG: Creep Fed AminoGut and later receiving a diet supplemented with AminoGut; SR: Sow reared pigs; PCNA: Proliferating cells nuclei antigen; SBM: Soy bean meal; AA: Amino acids; C: Celsius; CO2: Carbon dioxide; SEM: Scanning electron microscopy; HPLC: High performance liquid Chromatography; PAD: Pulsed amperometric detector; BW: Body weight; ADG: Average daily gain; mM: Millimolar; mL: Milliliters; nm: Nanometer; min: Minutes; μL: Microliters; ATP: Adenosine triphosphate; g: Gram; L: Liter; tRNA: Transfer ribonucleic acid; NAD: Nicotinamide adenine dinucleotide; NADP: Nicotinamide adenine dinucleotide phosphate; d: Day; mg: Milligram; kg: Kilogram; LPS: Lipopolysaccharides; TLR: Toll like receptors; NFĸB: Necrotic factor kappa B; CWG: Choice white grease; ZnO: Zinc oxide; NRC: National research council; ME: Metabolizable energy; CP: Crude protein; TID: Total ileal digestible.

## Competing interests

Full financial support for this trial was provided by Ajinomoto Heartland Lysine and Ajinomoto do Brazil. Dr. James L. Usry served as Swine Technical Director for Ajinomoto Heartland Lysine (currently serves as Swine Technical Director for Micronutrients), and both Marianne Kutschenko and Dr. Eduardo T. Nogueira serve as Technical Managers for Ajinomoto do Brazil. All three are authors in this manuscript. However their assistance was limited to guiding us on the design of the research protocol and the inclusion rate of both Glutamine and AminoGut. Both products are being evaluated but not currently being used in the US.

## Authors’ contributions

RAC as the lead author was in charge of designing the research protocol, obtaining all materials needed for it, executing it, carrying out all the lab work, data collection and analysis and finally writing the manuscript. JLU was instrumental on guiding the lead author on the design of the research protocol. CA provided the author with statistical guidance on the data analysis. ETN and MK provided significant literature and previous work references about glutamine and its biological functions as a non-essential amino acid. They were also involved in the design of the research protocol. JO as a co-advisor to the lead author, he was involved in the design, execution and interpretation of the results. He also assisted with blood and tissues collection and with the statistical analysis. AJM as a co-advisor to the lead author, he was involved in the design and interpretation of the results. He helped design our *in vivo* method for determining gut barrier function. All authors read and approved the final version of the manuscript.

## Authors’ information

RC holds a PhD in Animal Nutrition from North Carolina State University. His area of research is neonatal survival, nutrient digestibility and gastrointestinal health of swine. In 2001, he was awarded the “Innovative Award Applied Research” by National Pork Producer Council at the Midwest Animal Science Meeting in Des Moines, Iowa. He is a member of the North Carolina Pork Council and the American Society of Animal Science. He currently serves as Director of Swine Technical Services for Huvepharma USA, Inc. JLU holds a PhD in Agricultural Engineering from the University of Kentucky in animal growth modeling. He spent 21 years at Ajinomoto Heartland where he became VP of Nutritional Services and currently is employed at Micronutrients as Director of Swine Nutrition. Most of his career has centered on amino acid research and development. CA holds a PhD in Statistics from North Carolina State University. Her research interests include experimental design applied to life sciences, statistical modeling, and discrete data analysis. She is interested in statistical consulting, research methodology and creative learning and teaching. She is a Research Assistant professor in the Department of Statistics at NCSU and a member of American Statistical Society. ETN holds a PhD in Animal Nutrition from Viçosa Federal University (UFV, Brazil)/University of Western Australia (UWA, Australia). His area of research is amino acids nutrition. He currently serves as Latin America Technical General Manager for Ajinomoto do Brazil/Ajinomoto Animal Nutrition. MK holds an MSc in Animal Nutrition from Maringa State University (UEM, Brazil). Her area of research is amino acid nutrition. She currently serves as Latin America Technical Manager for Ajinomoto do Brazil/Ajinomoto Animal Nutrition.

AJM holds a MS in Swine Nutrition, a PhD in Gastrointestinal Physiology and a Doctor of Veterinary Medicine (DVM) all from NCSU. His main area of research is to study basic mechanisms of stress-induced intestinal dysfunction. Stress is an important contributing factor to enteric disorders of veterinary species and humans however, the mechanisms are poorly understood. His work has focused on the role of mucosal mast cells in psychological stress-induced disturbances in intestinal mucosal barrier function. He believes that this work will have important implications in the understanding of stress-related gut disorders such as infectious diarrhea, Inflammatory Bowel Disease, and Irritable Bowel Syndrome, and will facilitate the design of novel preventative and treatment strategies for veterinary and human patients suffering from these disorders. He is an assistant professor of GI physiology and swine medicine at NC State College of Veterinary Medicine. He is member of several professional societies including the American Physiological Society, American Association of Swine Veterinarians, and American Gastroenterological Association. JO has a PhD in Nutritional Biochemistry from the University of Wisconsin. As a Williams Neal Reynolds Professor in the Department of Animal Science at NCSU, his research interests are molecular and metabolic regulation of lipid digestion and metabolism; neonatal nutrition; intestinal growth and metabolism in normal and pathophysiological states. His program is focused on using the young piglet as a model for the human infant in nutrition and digestive physiology. His most recent awards include “Williams Neal Reynolds Distinguished Professor” and “The Outstanding Graduate Instructor” both given by the College of Agriculture and Life Science at NCSU, the “Animal Growth and Development Research” given by the American Society of Animal Science. He was a member of the National Research Council (NRC) committee which recently published the new 2012 Nutrient Requirements of Swine. He is an Associate Editor in Advances in Nutrition (American Society for Nutrition) and the Journal of Animal Science and Biotechnology.
